# Neighborhood Racial Segregation Predict the Spatial Distribution of Supermarkets and Grocery Stores Better than Socioeconomic Factors in Cleveland, Ohio: a Bayesian Spatial Approach

**DOI:** 10.1007/s40615-023-01669-4

**Published:** 2023-06-27

**Authors:** Ortis Yankey, Jay Lee, Rachel Gardenhire, Elaine Borawski

**Affiliations:** 1 WorldPop Research Group, School of Geography and Environmental Science, University Road, University of Southampton, Southampton, SO17 1BJ UK; 2 Department of Geography, Kent State University, 413 McGilvrey Hall, 325 S. Lincoln Street, Kent, OH 44240 USA; 3 Prevention Research Center for Healthy Neighborhoods, Case Western Reserve University, 11000 Cedar Ave, Cleveland, OH 44106 USA; 4 Department of Population & Quantitative Health Sciences and Nutrition, Case Western Reserve University, 2103 Cornell Road, Cleveland, OH 44106 USA

**Keywords:** Food deserts, Spatial analysis, Bayesian analysis, Neighborhood food environments, Racial segregation

## Abstract

**Introduction:**

The food environment influences the availability and affordability of food options for consumers in a given neighborhood. However, disparities in access to healthy food options exist, affecting Black and low-income communities disproportionately. This study investigated whether racial segregation predicted the spatial distribution of supermarkets and grocery stores better than socioeconomic factors or vice versa in Cleveland, Ohio.

**Method:**

The outcome measure was the count of supermarket and grocery stores in each census tract in Cleveland. They were combined with US census bureau data as covariates. We fitted four Bayesian spatial models. The first model was a baseline model with no covariates. The second model accounted for racial segregation alone. The third model looked at only socioeconomic factors, and the final model combined both racial and socioeconomic factors.

**Results:**

Overall model performance was better in the model that considered only racial segregation as a predictor of supermarkets and grocery stores (*DIC* = 476.29). There was 13% decrease in the number of stores for a census tract with a higher majority of Black people compared to areas with a lower number of Black people. Model 3 that considered only socioeconomic factors was less predictive of the retail outlets (*DIC* = 484.80).

**Conclusions:**

These findings lead to the conclusion that structural racism evidenced in policies like residential segregation has a significant influence on the spatial distribution of food retail in the city of Cleveland.

**Supplementary Information:**

The online version contains supplementary material available at 10.1007/s40615-023-01669-4.

## Introduction

The food environment is characterized by a variety of retail food establishments within a given neighborhood, such as supermarkets, grocery stores, and convenience stores, and many less conventional stores like gas stations, specialty food stores, and farmers markets. The retail food environment has a substantial effect on the availability and cost of food options for customers. Research findings indicate that some urban and rural residents live within a food desert where access to supermarkets and grocery stores is a challenge [1–4]. This problem is more pronounced among the urban poor and Black communities. Black et al. [3] presented a synthesis of peer-reviewed articles that examined disparities in the neighborhood food environment. They concluded that there is a widespread consensus that low-income residents and ethnic minority neighborhoods in the USA, such as Black neighborhood have disproportionately less access to healthy foods.

Inequality in access to supermarkets and large grocery stores has been conceptualized as being caused by two often overlapping factors: socioeconomic factors and racial segregation. The first posits that food deserts exist due to socioeconomic supply and demand constraints associated with the establishment of supermarkets and large grocery stores in urban and rural areas [5–7]. The high capital cost associated with the supply of supermarkets and the limited market potential for the demand for healthy food in low-income communities create a barrier to establishing grocery stores and supermarkets in such areas.

Moreover, supermarkets and large chain stores often demand more space than is available in many older, established, and more impoverished communities. Issues about land fragmentation, zoning regulations, a higher crime rate, and the general unattractiveness of these neighborhoods pose a supply constraint for supermarkets and larger retail store establishments in inner cities. For example, Bitler and Haider [6] discuss how zoning restrictions, crime, and other factors discourage the entry of supermarkets and grocery stores that would sell healthy foods in low-income areas.

Furthermore, a declining population in urban areas, low demand for healthy food items, a higher poverty rate, and a higher rate of adoption of income support programs such as the Supplementary Income Assistance Program (SNAP) and the Women, Infants, and Children (WICS) nutritional programs pose an effective demand constraint for supermarket and larger chain store establishments in urban areas [5, 6]. For example, Allcott et al. [7] investigated the causes of nutritional inequality between high- and low-income households in the USA. They discovered that even after establishing a new supermarket in a low-income neighborhood, demand for healthy grocery purchases was relatively low for low-income households compared to high-income households. They concluded that the entry of a new supermarket has an economically small effect on healthy grocery purchases and that nutritional inequality is more of a demand problem.

An alternative perspective on the food desert problem is that racial segregation is a fundamental cause of inequality in the availability of healthy food options [8, 9]. Racial segregation has long been established as the foundation for Black and White health inequalities [10]. Structural factors exemplified in social and economic systems have rendered some low-income and predominantly Black neighborhoods unattractive to the establishment of large-scale supermarkets and grocery stores. Pervasive housing policies that discriminated against predominantly Black populations, such as the redlining of cities that started with the establishment of the Homeowners’ Loan Corporation in the 1930s, generated a vicious cycle of disinvestment in inner cities and the suburbanization of higher-income Americans (mostly White populations). The suburbanization of wealthier urban residents further resulted in the flight of supermarkets and grocery stores from the central cities to these suburbs, as the suburbs were seen as a vibrant investment opportunity [11].

For instance, Thibodeaux [12] examined the evolution of the supermarket landscape between 1970 and 1990. He found that in 1970, supermarkets were more likely to locate in urban areas with higher poverty and lower income. However, this pattern of supermarket location gradually shifted their focus to the suburbs from 1970 to 1990 as White and more affluent urban residents moved to the suburbs. Similarly, Alwitt and Donley cited in Walker [2] stated that the flight of middle- and higher-income urban residents in the 1970s and 1980s from the inner city towards the suburbs caused the median income in the inner cities to significantly decline, forcing nearly one-half of the supermarkets in the three largest US cities to close. Currently, there are food retail gaps, with Black neighborhoods underserved by large-scale supermarkets and grocery stores.

Racism and socioeconomic status are two distinct yet interconnected mechanisms that are mutually reinforcing. The intertwined nature of racism and socioeconomic status serves to reinforce inequality in both health outcomes and the built environment, particularly among Black populations [10, 13]. The theory of systemic racism, as described by Feagin [14, 15], identifies several key features of racism in the USA, including the prioritization of whiteness and the perpetuation of racial and material disparities throughout society. He argues that for over 20 generations, White people have inherited economic resources unfairly acquired through racial oppression such as slavery and segregation, resulting in the impoverishment of people of color, particularly Black people. Today, systemic and institutional discrimination and the unjust inheritance of resources continue to limit opportunities for people of color, including access to quality education, healthcare, employment, and political power. These disparities can exacerbate poverty, poor health outcomes, and social exclusion, creating a vicious cycle where low socioeconomic status increases exposure to various forms of discrimination, including racism, and heightens the risk of poor health outcomes. Thus, addressing systemic racism and socioeconomic inequality must be key components of any effort to promote health equity and social justice.

Although racial segregation and socioeconomic factors have been suggested as two driving factors for the inequality in supermarket and grocery store access in urban areas, we sought to explore and better understand the differential effect of these two factors on supermarket and grocery store access. While economic factors and racial segregation are inextricably linked, understanding how each of these factors affects the food environment may provide valuable insights into the urban food environment, potentially suggesting differential policy implications and directions for mitigating food desert challenges.

Also, a relatively unexplored problem is the relationship between measuring and operationalizing residential segregation and its effect on retail outlet density. Residential segregation and food desert studies have frequently relied on simplistic indicators of neighborhood segregation, such as the percentage of Blacks in an area. It is well established that segregation follows or manifests itself in a variety of spatial patterns, and several formal indices related to evenness, exposure, concentration, clustering, and centralization exist to quantify residential segregation [16–18]. Measuring racial segregation using formal segregation indices is therefore crucial for elucidating disparities in retail food access, which our study sought to explore. Finally, many food environment studies adopt a non-spatial method that ignores the spatial correlation of observations and neighborhood covariates across geographic space, leading to a potential bias in the parameter estimates.

In this regard, we use formalized segregation indices related to evenness and clustering [16] to examine disparities in the retail food environment using a Bayesian spatial method that accounts for spatial correlation in the observed variables under study. We also disaggregate socioeconomic factors from neighborhood segregation factors in two separate models and test the research question: Does racial segregation predict the spatial distribution of supermarkets and grocery stores much better than socioeconomic factors or vice versa in the city of Cleveland?

The city of Cleveland, Ohio, is used as the study location to examine the spatial distribution of supermarkets and grocery stores. Cleveland historically had a pervasive racial segregation policy, including the redlining of the city. Currently, Cleveland ranks between 5th and 10th as the most racially segregated city in the USA, depending on the method used [19, 20]. This has affected every facet of the city’s urban landscape. The level of racial segregation in Cleveland is ubiquitous and glaring, with a very sharp contrast between neighborhoods with a majority White population and neighborhoods with Black population. Cleveland is also one of the poorest cities in America, with the majority of its neighborhoods being low-income but segregated by race.

## Methods

### Outcome Variable: Food Retail Data

Food retail outlet data for the city of Cleveland was obtained from the Prevention Research Center for Healthy Neighborhoods (PRCHN) at Case Western Reserve University School of Medicine. As part of the Neighborhood Environmental Assessment Project (https://prchn.org/neap/), the PRCHN conducts an annual food retail audit of every food establishment in the city of Cleveland and selected suburbs. The audit is a field survey that collects data about the store, the products it sells, and whether the store is open or closed. Using a standardized audit tool, the program assesses the availability of over 25 food items, with a specific interest in foods supported by nutrition assistance programs such as WIC and SNAP. These food items are then used to classify food stores based on the availability of a variety of staple food items that support a healthy lifestyle [21]. The PRCHN classification is hierarchical, with supermarkets representing the greatest variety of healthy lifestyle foods and corner or convenience stores selling at least one food item but very few staple food options (e.g., milk, bread, eggs). The PRCHN project began in 2012, and data are collected during the summer months.

We used the 2019 dataset for this study and focused only on supermarkets, large grocery stores, and small grocery stores, as they are the ones that carry most of the basic staples needed to prepare food at home. We combine supermarkets, large grocery stores, and small grocery stores into a single category for the analysis. The stores were combined as one outcome for the purpose of this study because most studies on the food environment use these stores as a proxy for determining healthy food stores within a community. The outcome was the count of these stores in each census tract. Stores immediately outside the geographic boundary of the city of Cleveland were also included in the analysis to account for edge effect since these stores were equally accessible by communities living on the periphery of the city boundary.

### Predictor Variables: Racial Composition and Socioeconomic Covariates

Racial composition and socioeconomic characteristics data were used as predictor variables. The data was obtained from the US Census Bureau. These variables were based on the US Census Bureau’s 5-year estimate (2014–2018), which has the advantage of increased statistical reliability compared to the 1-year estimates [22]. The data was based on census tract estimates.

### Racial Segregation Measures

A multi-racial entropy index was calculated using White populations, Black/African American populations, Asian populations, and Hispanic populations to measure racial segregation [16, 23, 24]. The entropy index is an evenness residential segregation index that measures each census tract’s weighted average deviation from the city’s racial or ethnic diversity. The values of an entropy index range between 0 and 1. A value of 0 means that all census tracts have the same composition as the entire city (integration), and a value of 1 means that all census tracts contain only one racial group (segregation) [16, 23]. We also accounted for the clustering of the White population, the clustering of the Black population, the percentage of the Asian population, and the percentage of the Hispanic population. A local G-Statistics [25] was used in calculating the clustering of the White population and Black population. Based on the local G-Statistics, census tracts with a positive z-value were classified as a high cluster for both Black and White populations, and census tracts with a negative z-value were classified as a low cluster for both populations.

We used a multi-racial entropy index because, while most segregation measures account for segregation between only two or even one group, such as clustering measures, the multi-racial entropy index was used to measure segregation between multiple groups [23]. This is important because segregation manifests itself in diverse forms within an urban environment, and hence, the entropy index was used to provide a holistic approach of combining multiple races (White population, Black/African American population, Asian population, and Hispanic population) in a single segregation measure, unlike the clustering approach, which measures only a single racial composition.

### Socioeconomic Variables

The percentage of households below poverty levels, the percentage of SNAP recipients, the percentage of households with no vehicle, the percentage of vacant housing, and the percentage of college-educated people were used as socioeconomic variables to answer the question of whether socioeconomic factors predict the spatial distribution of the stores. We selected these variables based on the literature that examines the demand and supply constraints of supermarkets and grocery stores discussed in the introduction. For instance, the percentage of people below poverty was used as a proxy to measure effective demand (market potential) for the stores [5, 6]. The percentage of vacant housing, for example, is also a supply constraint because an urban blight neighborhood becomes unattractive for investors in such large-scale stores.

Before performing any analysis, we checked the multicollinearity of the predictor variables using the variance inflation factor (VIF). A high VIF value is an indication of multicollinearity among the predictor variables. A high VIF is defined subjectively; however, we used a VIF value of 6 as the threshold to measure multicollinearity [26]. None of our predictor variables exceeded this threshold. Therefore, multicollinearity was minimized in our study.

### Analytical Strategy

A hierarchical Bayesian spatial model was used to examine the spatial distribution of the retail outlet. A Bayesian spatial model includes a spatial random effect term into the model to account for spatial dependence between adjacent census tracts that is not explained by the model covariates. [27, 28]. Models that incorporate spatial dependence in the model have been found to be statistically robust compared to non-spatial methods when dealing with spatial data [29].

Bayesian models can be implemented by simulating the posterior estimate using the Markov chain Monte Carlo (MCMC) method [30]. However, MCMC is computationally time consuming when dealing with a large spatial dataset. Hence, a more deterministic model that approximates the posterior estimate called the Integrated Nested Laplace Approximation (INLA) offers a computationally faster algorithm than MCMC [31, 32]. In this study, we used INLA for the analysis using the R-INLA Package.

### Model Specifications

We estimated the observed count of the stores as a function of the covariates using a zero-inflated Poisson (ZIP) model to account for overdispersion in the modeling due to the excessive number of zero store counts in some census tracts. Because the outcome variable is count, a log-link function was used to express the set of covariates given as a function of the outcome variable. The model is expressed in linear form as:

$$\log \left(\ {\lambda}_i\right)={\beta}_0+{\beta}_1{X}_{i1}+{\beta}_2{X}_{i2}+\dots +{\beta}_n{X}_{in}+{u}_i+{v}_i+ ei,i=1,2,\dots, n,$$

where *β*_0_ is the intercept, β’s are the regression coefficients for the covariates, *u*_*i*_ is spatially structured effect to account for spatial dependency in the distribution of the food retail outlet among the census tracts, *v*_*i*_ is spatially unstructured random effect to account for uncorrelated random errors, and *ei* is an offset term that is given as the log of store count standardized by total population in each census tract. The spatially structured component *u*_*i*_ is modeled as an intrinsic conditional autoregressive (ICAR) process proposed by Besag et al. [33], which smooths the spatial effect based on a certain neighborhood definition. The unstructured component *v*_*i*_ is modeled as independent and identically distributed normal variables with zero mean and variance ${\sigma}_v^2$. We specified four models in this study.

### Model 1

The first model was an intercept (baseline) model whereby only spatial effect explained the count of supermarkets and grocery stores. The spatial effect is decomposed into two components, namely a spatially structured effect and a spatially unstructured effect. The model is given as:

$$\log \left(\ {\lambda}_i\right)={\beta}_0+{u}_i+{v}_i+ ei$$

where the model assumes the usual notation as defined above. This model is a baseline model with no covariates.

### Model 2

In model 2, we considered racial segregation as the only covariates. This model was used to evaluate whether racial segregation explained the spatial distribution of the supermarkets and grocery stores observed in the city of Cleveland. The model is given by:

$$\log \left(\ {\lambda}_i\right)={\beta}_0+{X}_n\beta +{u}_i+{v}_i+ ei$$

where the equation assumes the usual notations in model 1 and *β*^’^s is the coefficient of the covariates *X*_*n*_. The covariates considered in this model include the following: *X*_*n*_*β* = [*β*_1_(*Entropy Index*) + *β*_2_(*Black Clustering*) + *β*_3_(*White Clustering*) + *β*_4_(%*Asian*) + *β*_5_(%*Hispanic*)]

### Model 3

In the third model, we reparametrized the first model by incorporating socioeconomic factors as the only covariates in the model. This model was used to evaluate whether socioeconomic factors explained the spatial distribution of the food stores:

The model is given by:

$$\log \left(\ {\lambda}_i\right)={\beta}_0+{X}_n\beta +{u}_i+{v}_i+ ei$$

where: $${X}_{n}\beta =\left[{\beta}_{1}\left(\% Below\ Poverty\right)+{\beta}_{2}\left(\% SNAP\ Recepient\right)+{\beta}_{3}\left(\% Household\ No\ Vehicle\right)+{\beta}_{4}\left(\% Vacant\ Housing\right)+{\beta}_{5}\left(\% College\ Educated\right)\right]$$

### Model 4

The fourth model is a full model and incorporates all the covariates in the modeling. The model is given by:

$$\log \left(\ {\lambda}_i\right)={\beta}_0+{X}_n\beta +{u}_i+{v}_i+ ei$$

where the equation assumes the usual notation in the previous models and:

$${X}_{n}\beta =\left[{\beta}_{1}\left( Entropy\ Index\right)+{\beta}_{2}\left( Black\ Clustering\right)+{\beta}_{3}\left( White\ Clustering\right)+{\beta}_{4}\left(\% Asian\right)+{\beta}_{5}\left(\% Hispanic\right)+{\beta}_{6}\left(\% Below\ Poverty\right)+{\beta}_{7}\left(\% SNAP\ Recepient\right)+{\beta}_{8}\left(\% Household\ No\ Vehicle\right)+{\beta}_{9}\left(\% Vacant\ Housing\right)+{\beta}_{10}\left(\% College\ Educated\right)\right]$$

### Prior Choice and Model Reporting

Bayesian models rely on prior distributions to reflect the state of our knowledge on the potential distribution of the parameter of interest. In this study, we resorted to the use of default priors in INLA, which are all non-informative priors. The priors are given by their mean and precision. The following prior distributions were assumed for the regression parameters:

$${\beta}_0\sim Normal\left(0,0.001\right)$$

$${\beta}_i\sim Normal\left(0,0.001\right)$$

And the spatial random effect terms *u*_*i*_ *and v*_*i*_ were assigned a logGamma prior with a mean of 1 and precision *τ* = 0.00005:

$$\log\;\tau_{u_i}\sim\log\;Gamma\left(1,0.00005\right),$$

$$\log\;\;\tau_{v_i}\sim\log\;Gamma\left(1,0.00005\right)$$

The reason why we used the default priors in INLA is that these priors are well-established and widely used in the literature for similar spatial modeling techniques and have been found to perform best [34, 35]. The posterior distribution of the four model parameters was summarized using the mean and the 95% credible interval (CI). The 95% CI was used to determine the statistical significance of the posterior mean of the various variables considered in each model. A variable is considered statistically significant if the interval does not contain a value of 1. Also, we used the deviance information criterion (DIC) to evaluate the performance of the four different models. A lower DIC value signifies a better model fit coupled with model parsimony.

We also examined the exceedance probabilities from the model to determine census tracts with a higher count of stores after accounting for the model covariates. The exceedance probabilities refer to the probability that a parameter of interest exceeds a given threshold. The exceedance probability that a parameter *λ* exceeds or is greater than a given threshold c is given as P ( *λ* > *c*|*data*). In this study, we used 1 (exp0) as the threshold to determine locations where store counts were higher or lower after accounting for model covariates. Bayesian exceedance probabilities have been proposed as a Bayesian approach to hotspot/coldspot identification [27]. The exceedance probabilities range between a low value of 0 and a high value of 1. Richardson et al. [36] provide a categorization of the exceedance probabilities into hotspots and coldspots. Exceedance probabilities between 0 and 0.2 are considered a coldspot, 0.2–0.8 are neither coldspots nor hotspot, and 0.8 to 1 are considered a hotspot. We use this classification to determine the spatial distribution of the stores after accounting for the model covariates.

## Results

### Descriptive Statistics

**Fig. 1:**
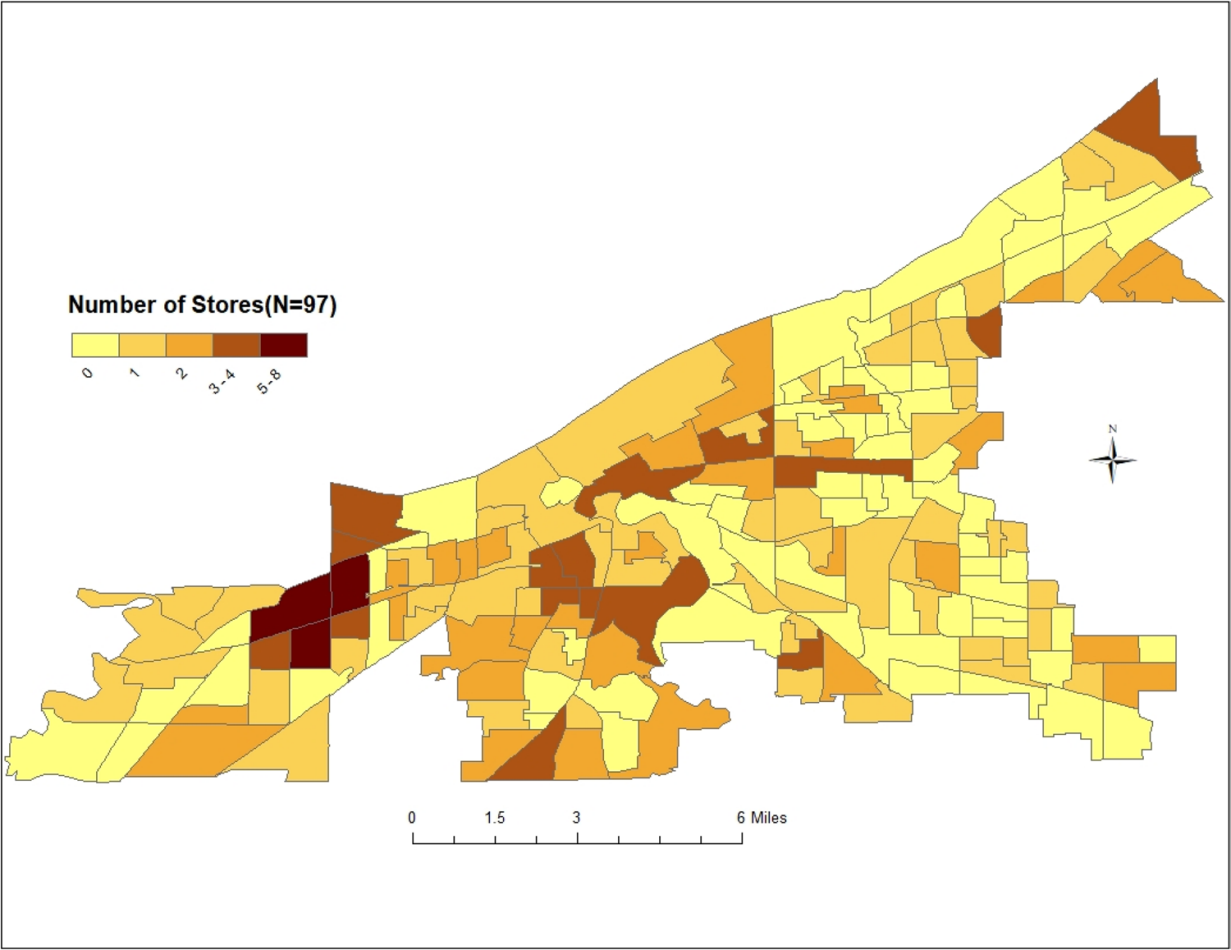
Number of stores per census tract

Figure 1 shows the spatial distribution of the stores (supermarkets, small grocery stores, and large grocery stores) in the study location. The number of stores varies considerably across census tracts in the city. Census tracts with no supermarkets or grocery stores (zero count) are given in a light-yellow color, and these tracts are largely found in the eastern portion of the city. Areas with a higher number of these stores are color-coded “deep brown,” and they are found in the center and western sections of the city. Table 1 presents descriptive statistics for the variables considered in fitting the models. The mean entropy index was 0.52 with a standard deviation of 0.28, indicating that, on average, Cleveland is a highly segregated city. The mean percentage of households below poverty levels was 36.37%. Higher clustering of the Black population was found in 56.50% of the census tracts, whereas 43.50% of the census tracts had low Black clustering. Higher White clustering was found in 50.80% of the census tracts, whereas 49.20% of the census tracts had low White clustering. The city has a relatively low mean percentage of Hispanics (10.07%) and Asian populations (2.19%). The mean percentage of SNAP recipients was 36.76%, with a standard deviation of 15.64%. The mean percentage of households with no vehicle was 25.54%, and vacant housing was 21.51%, The VIF showed that all the variables were below the threshold level of 6. Hence, multicollinearity was minimal. The spatial distribution of the study covariates has been provided as supplementary material (Fig. 4).

**Table 1:** Descriptive statistics for study variables used in examining the spatial distribution of store count (*N* = 97)

	Mean (percent)	Std. Dev	Minimum	Maximum	VIF
Entropy index	0.52	0.33	0.00	1.00	5.38
Percentage — population below poverty	36.37	14.96	0.00	89.20	4.06
Clustering — Black population					5.79
High clustering	56.50%				
Low clustering (reference)	43.50%				
Clustering — White population					5.45
High clustering	50.80%				
Low clustering (reference)	49.20%				1.94
Percentage — Hispanic	10.07	13.00	0.00	55.90	4.46
Percentage — household SNAP Recipient	36.76	15.64	0.00	91.00	5.27
Percentage — household with no vehicle	25.54	14.74	0.00	77.40	2.36
Percentage — vacant housing	21.51	10.37	0.00	48.40	1.68
Percentage — college educated	9.46	7.18	0.00	41.10	2.05

The continuous predictors are presented using their mean value and standard deviation whereas the categorical predictors are presented as percentages. The two categorical variables are the clustering of Black population and clustering of White population. The VIF column shows the variance inflation factor for each predictor, which measures the degree of multicollinearity in the predictors. A lesser VIF value indicates multicollinearity is minimal

### Association Between Study Variables and the Food Retail Outlets

In assessing the association between the study variables and the food retail outlets, the posterior distribution of the coefficients from the various models is summarized using the mean and their corresponding 95% credible interval (CI). Rather than a single point statistic associated with frequentist models, Bayesian statistics summarize the parameter of interest using a posterior distribution, which encompasses the entire possible range of values for the parameter of interest. The posterior distribution can then be summarized using the mean or median value. The posterior distribution from the various models can be found in the supplementary materials.

Table 2 presents the posterior mean (model-based rate ratio, RR) and the corresponding 95% credible interval (CI) for all the models. Model 1 is an intercept-only model with no covariates. This model was used as a baseline to assess the performance of the other models. There is only one fixed effect in this model, which is the intercept. The posterior mean (rate ratio) value for the intercept in model 1 is 1.06 (95% CI 0.87–1.30). This represents the average count of supermarkets and grocery stores across the census tract.

**Table 2:** Posterior estimates for the association between store count and racial/socioeconomic covariates (*N*=97)

Fixed effect	Model 1		Model 2		Model 3		Model 4	
	Adjusted effect		Adjusted effect		Adjusted effect		Adjusted effect	
	Mean (RR)	95% CI	Mean (RR)	95% CI	Mean (RR)	95% CI	Mean (RR)	95% CI
Intercept	1.06	0.87–1.30	0.78	0.42–1.47	1.01	0.48–2.06	0.99	0.39–2.49
Entropy index			0.61	0.18–2.06			0.55	0.15–2.01
High White clustering (Ref=low)			1.92	0.97–3.85			1.86	0.86–4.08
High Black clustering (Ref=low)			0.87*	0.55–0.98			0.87	0.44–1.74
Percentage — Asian population			1.02	0.99–1.05			1.01	0.97–1.05
Percentage — Hispanic			1.02*	1.00–1.03			1.02*	1.00–1.04
Percentage — population below poverty					1.01	0.99–1.03	1.01	0.99–1.03
Percentage — household SNAP recipient					0.98*	0.96–0.99	0.98	0.96–1.01
Percentage — vacant housing					0.99	0.98–1.02	0.98	0.96–1.02
Percentage — household with no vehicle					1.01	0.99–1.02	1.01	0.99–1.03
Percentage — college educated					0.99	0.96–1.02	0.99	0.96–1.02
Random effect								
Spatially structured effect	4.56	1.10–2.07	0	0.00–3.88	1.02	0.00–2.04	4.37	0.09–5.35
Spatially unstructured effect	0	0.00–1.67	0	0.00–1.79	1.72	0.00–1.50	5.26	0.00–8.35

The table shows the posterior estimates of the adjusted effects of the covariates on the store count, using four different Bayesian spatial models. The mean (RR) column shows the mean of the posterior distribution of the rate ratio (RR) for each covariate, and the 95% CI column shows the 95% credible interval of the RR. An RR above 1 indicates a positive association between the covariate and the store count, while an RR below 1 indicates a negative association. An asterisk (*) indicates that the 95% credible interval does not include 1, which suggests a statistically significant association. The random effect shows the posterior estimates of the spatially structured and unstructured effects, which capture the spatial dependence in the store count due to the census tracts neighboring structure

Model 2 reports the effect of racial segregation on the count of stores. We found that the entropy index was negatively associated with the count of healthy food retail, with a 39% (*RR* = 0.61; 95% CI: 0.18–2.06) decrease in the number of supermarkets and grocery stores as the level of segregation increased. Areas with a higher White clustered population had a 92% (*RR* = 1.92, 95% CI: 0.97–3.85) increase in the number of stores relative to low White clustered areas; however, this was not statistically significant. Higher Black clustered areas had a 13% (*RR* = 0.87, 95% CI: 0.55–0.98) decrease in the number of stores relative to low Black clustered tracts. This result was statistically significant. Furthermore, we found a statistically significant positive association between the percentage of Hispanics and the number of stores, with a 2% increase in the number of stores as the percentage of Hispanics increased (*RR* = 1.02, 95% CI: 1.00–1.03). Similarly, a percentage increase in the Asian population was associated with a 2% increase in the number of stores (*RR* = 1.02, 95% CI: 0.99–1.05); however, this was not statistically significant.

Model 3 reports the effect of socioeconomic factors on the number of supermarkets and grocery stores. The adjusted effect shows that the percentage of families below poverty was associated with a 1% increase in the number of supermarkets and grocery stores (*RR* = 1.01; 95% CI: 0.99–1.03). Households without a private car were also associated with a 1% increase in the number of supermarkets and grocery stores (*RR* = 1.01; 95% CI: 0.99–1.02). On the contrary, the percentage of SNAP recipients (*RR* = 0.98, 95% CI: 0.96–0.99), vacant housing (*RR* = 0.99, 95% CI: 0.98–1.02), and the percentage of college-educated people (*RR* = 0.99, 95% CI: 0.96–1.02) were negatively associated with the number of supermarkets and grocery stores.

Model 4 is the accumulative model, which includes the adjusted effects of both segregation and socioeconomic factors on the distribution of supermarkets and grocery stores. We found that the effects of the entropy index (*RR* = 0.55, 95% CI: 0.15–2.01), high Black clustering (*RR* = 1.86, 95% CI: 0.86–4.08), percentage of SNAP recipients (*RR* = 0.98, 95% CI: 0.96–1.01), vacant housing (*RR* = 0.98, 95% CI: 0.98–1.02), and college-educated (*RR* = 0.99, 95% CI: 0.96–1.02) were negatively associated with the density of the stores. On the contrary, we see a positive association for areas with a high White clustered population when compared to areas with a low White clustered population (*RR* =1.86, 95% CI: 0.86–4.08), the percentage of Asian population (*RR* = 1.01, 95% CI: 0.97–105), the percentage of people below poverty (*RR* = 1.01, 95% CI: 0.99–1.03), and the percentage of Hispanic population (*RR* = 1.01, 95% CI:1.00–1.03). The percentage of the Hispanic population had a statistically significant result.

### Models Assessment

The deviance information criterion (DIC) was used to measure the performance of the various models as presented in Table 3. There is no formal test for comparing DIC values for two models; however, differences in two DIC values ranging between 3 and 7 have been proposed to demonstrate sufficient evidence that the model with the smaller DIC fits better than the alternative model [37, 38]. All the models were compared to model 1 (which was the baseline model without covariates). Comparing all the models, model 2 (which examined racial segregation) was the best model with a DIC value of 476.29. This was a 6-point drop in the DIC relative to model 1 (*DIC* = 481.87). Model 3 (*DIC* = 484.90) and model 4 (*DIC* = 483.04) were less predictive of supermarket and grocery store distribution in the city. This means that although racial segregation and socioeconomic factors are two complementary factors that are associated with the food environment, the spatial distribution of supermarkets and grocery stores in Cleveland is better explained by racial segregation than socioeconomic factors.

**Table 3:** Model diagnostics

	Effective number of parameters (pD)	Deviance information criterion (DIC)
Model 1	14.44	481.87
Model 2	6.78	476.29
Model 3	22.61	484.90
Model 4	11.77	483.04

The table shows the model diagnostics for the four regression models. The effective number of parameters (pD) column shows the measure of model complexity, which accounts for both the fixed and random effects. The deviance information criterion (DIC) column shows the measure of model fit, which balances the model complexity and the deviance. A lower pD or DIC value indicates a better model. Based on these criteria, model 2 has the best fit among the four models

### Spatial Effect of Supermarkets and Grocery Store Hotspot Analysis

**Fig. 2:**
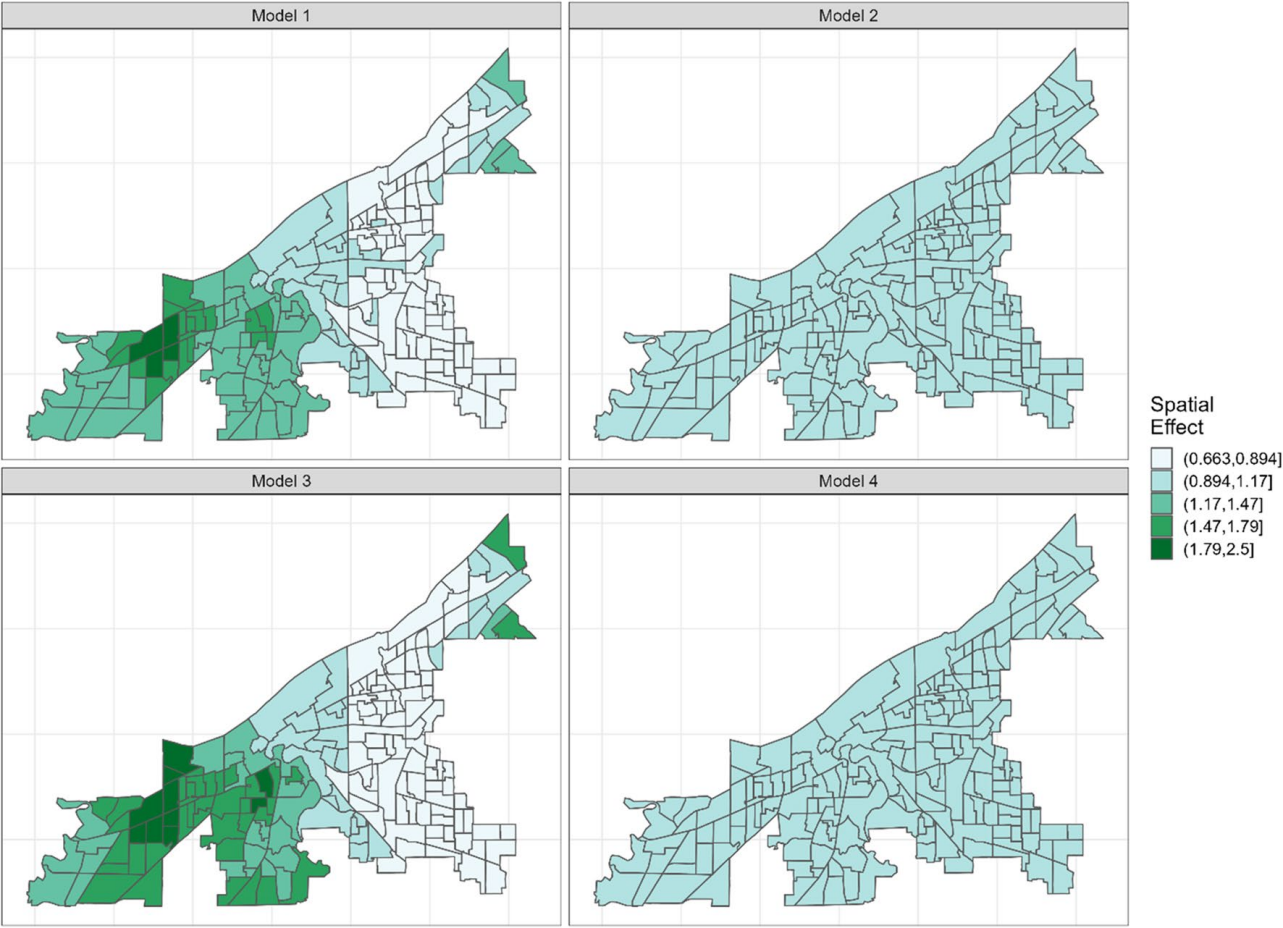
Showing the posterior mean for census tract specific rate ratio

**Fig. 3:**
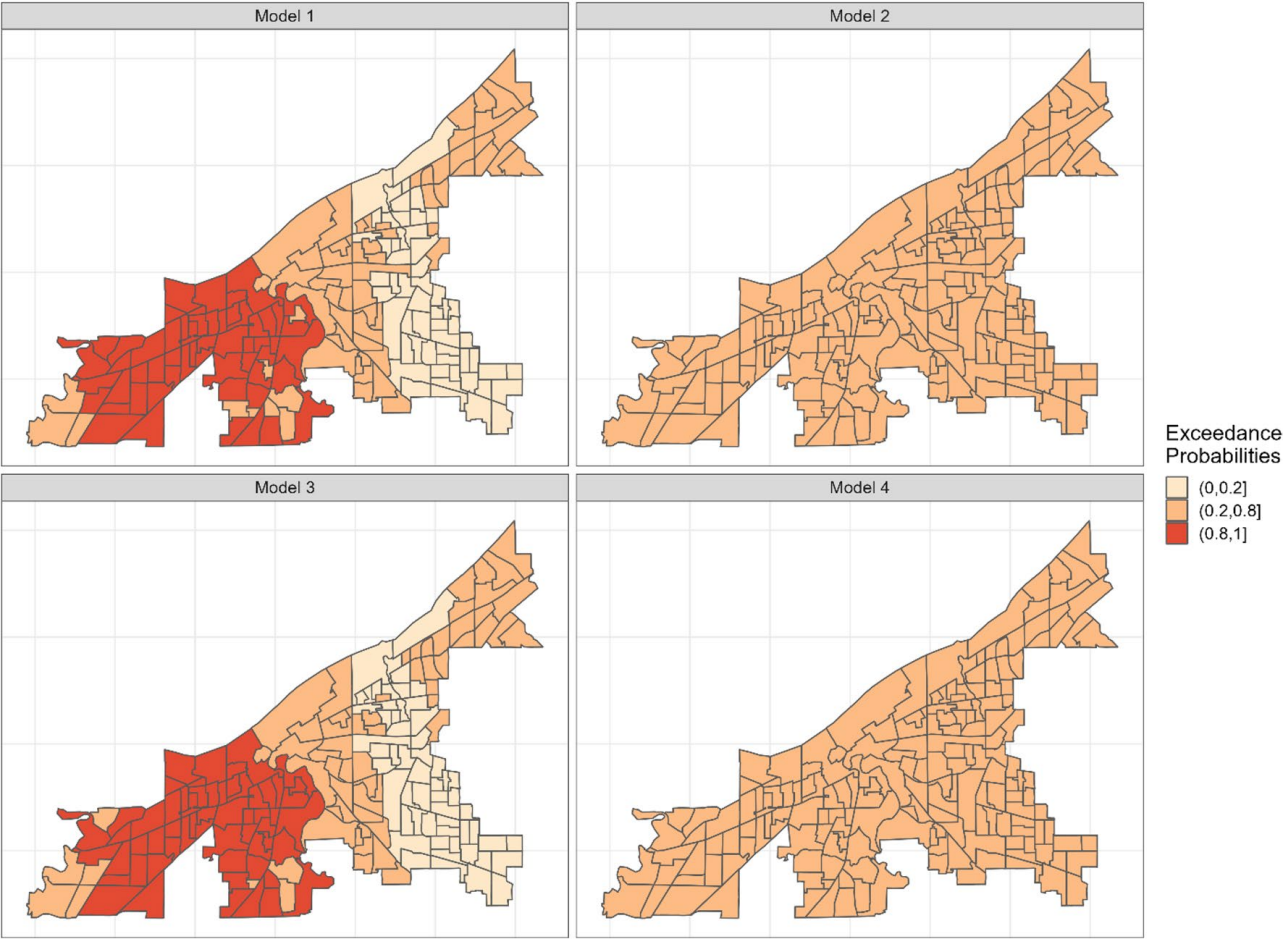
Showing the posterior exceedance probabilities

Figure 2 show the spatial effect of the retail outlets. The spatial effect explains variations in the outcome variable attributable to spatial processes that the model’s covariates do not account for. The posterior estimates from the spatial effect component show areas where the count of stores is either higher or lower than the average count of stores across the city after accounting for the covariates in the various models. The posterior estimates from models 1 and 3 are relatively similar to each other, whereas models 2 and 4 are also similar to each other. The posteriors from the spatial effect were examined to see if they exceeded a given threshold; in this case, 1 (exp0) was used as the threshold. The exceedance probabilities were classified using the classification proposed by Richardson et al. [36]. The exceedance probabilities are presented in Fig. 3. In models 1 and 3, areas to the west have a higher exceedance probability (above 0.8) after accounting for the models’ covariates, indicating that these areas have a higher number of stores relative to the city’s average. In models 2 and 4, the number of stores can be said to be moderately distributed across the city, based on the exceedance probability value that fell between 0.2 and 0.8.

## Discussions

Cleveland has been identified as a “food desert,” with many of its urban residents living beyond the immediate reach of supermarkets and grocery stores [39]. In this study, we disaggregated racial segregation and socioeconomic factors to examine whether racial segregation and socioeconomic factors have independent or overlapping relationships with the number of supermarkets and grocery stores in a predominantly low-income community using four spatial Bayesian models.

The results of our study suggest that contrary to the economic hypothesis that food deserts are created as a result of supply and demand constraints associated with socioeconomic factors [5–7], racial segregation as a fundamental cause of food deserts [8, 9] explains the spatial distribution of supermarkets and grocery stores in Cleveland, Ohio. Overall model performance was better in model 2, which considered only racial segregation variables as predictors of the spatial distribution of supermarkets and grocery stores in the city of Cleveland, compared to model 3, which examined only socioeconomic factors, or model 4, which combined all the variables in the analysis. Particularly, we found that Black clustered census tracts had a lesser number of supermarkets and grocery stores compared to White clustered neighborhoods. This result supports the argument that systematic racism that affects Blacks more than other races underpins food access in American cities [8, 40].

Feagins et al. [14, 15] contend that structural racism manifests in the form of limited access to resources, which subsequently influences the built environment. The authors argue that institutional discrimination and structural racism impede the availability of resources in Black communities, such as access to quality education, healthcare, employment opportunities, and political power. Within the context of the food environment, racial segregation policies have contributed to the uneven distribution of healthy food options in urban areas [8, 9, 40–42]. For example, a survey of the retail environment in 39 US cities reported that retail food outlets systematically underserved neighborhoods with high percentages of African Americans but not Latino and lower-income non-African American groups, leading to the conclusion that the inner-city retail gap is in part racial in nature [41]. This has resulted in Black neighborhoods having limited access to healthy retail options while being exposed to unhealthy eating behaviors through the establishment of fast-food restaurants [43, 44], alcohol outlets [45], and other unhealthy retail outlets, leading to poor health outcomes among Black communities. According to Williams [10], residential segregation is the “single most important [land use] policy” that continues to adversely affect the socioeconomic status and health of African Americans.

Responses to unequal food access have often involved a simplistic market intervention policy, such as the Healthy Food Financing Initiative (HFFI), aimed at establishing new supermarkets and grocery stores in underserved areas. The notion is that when these stores are built, Black residents will patronize them, and the stores will profit in the long run. However, some research has found that some of these policies have done little to improve the food environment, healthy eating behavior, or overall health impact [46, 47]. Worse yet, some of these stores have closed altogether since their establishment [48]. The usual economic hypothesis for supermarket and grocery store failure in inner cities has been that of low demand for the stores, hence their failure [5–7]. Thus, effective demand to sustain supermarkets and large grocery stores in Black and low-income communities is limited and the owners of these supermarkets and grocery stores are capitalists who see themselves less in the business of feeding the poor and more in the business of making profits. Hence, supermarkets and large grocery stores prefer to establish themselves in White and affluent neighborhoods.

Such a narrow economic perspective of supermarket failure in inner cities fails to also consider systemic racism in the built environment, particularly the redlining of cities, which led to the flight of supermarkets and large grocery stores to largely White communities [11, 12]. This may be a reason why we have a low count of grocery stores in Black clustered census tracts in Cleveland, Ohio, compared to White clustered census tracts, as has been found in this study. Secondly, other researchers such as Howerton and Trauger [49] have also found that stereotypical negative place narratives and race-based fears about the location of a store lead to low patronage by outsiders, eventually leading to the collapse of a store due to low-profit margins. This reason may also suggest the low number of supermarkets and grocery stores in Black communities within the city of Cleveland.

Although racial segregation is a better predictor of the count of supermarkets and grocery stores in Cleveland, this does not imply that socioeconomic factors are irrelevant in explaining the food environment in Cleveland. Socioeconomic factors such as income, education, and employment status are often associated with racial segregation [8, 50] and can act as mediators in the relationship between racial segregation and the distribution of grocery stores. For example, in a highly segregated community with predominantly low-income residents, there may be fewer grocery stores due to lower demand for healthy food options and a lack of investment in the community by grocery store chains [5–7]. In this case, the effect of racial segregation on the distribution of grocery stores may be mediated by socioeconomic factors such as income and employment status. This could be a reason why the result from model 4, which looked at both socioeconomic and racial factors combined, was less predictive of the stores in Cleveland.

The limitations of this study include our inability to draw causal inferences between the predictors and the food retail count due to the use of cross-sectional data. Another limitation has to do with using census tracts to define neighborhoods in the study context. City neighborhoods, in reality, are different from census tracts. Also, census tracts are subject to the modifiable area unit problem, whereby the aggregation of data at a larger spatial scale, such as the census tract, may produce statistical bias leading to generalization across a larger spatial scale with inherent heterogeneity. Notwithstanding such limitations, our study is significant in terms of the methodology we utilized. Even though the food environment has been widely researched, relatively few studies have adopted a Bayesian method in their analysis, such as Luan et al. [51] and Lamichhane et al. [52]. Our study adds to the recent literature that explores the food retail environment using Bayesian models. Also, the retail food dataset and the classification of the stores used in this study were based on an actual ground-truthing survey to collect data on these stores. Many food retail studies have relied on secondary data sources that are subject to limited data validity, misclassification, and geocoding errors compared to primary surveys [53].

## Conclusions

Guided by the findings in this research, this research argues that structural factors exemplified by pervasive segregationist policies through housing discrimination and redlining have affected every facet of Black communities, including the food environment. This study supports the hypothesis that racial segregation is a fundamental cause of food deserts in the city of Cleveland. Slocum and Saldanha [42] sum it up perfectly when they state that “racism is endemic to global food systems in the aftermath of colonialism, with predictable results for minority communities.”

### Supplementary information

ESM 1: (DOCX 1519 kb)

## References

[1] Cummins S, Macintyre S (2002). “Food deserts”—evidence and assumption in health policy making. Bmj..

[2] Walker RE, Keane CR, Burke JG (2010). Disparities and access to healthy food in the United States: a review of food deserts literature. Health Place.

[3] Black C, Moon G, Baird J (2014). Dietary inequalities: what is the evidence for the effect of the neighbourhood food environment?. Health Place.

[4] Bower KM, Thorpe RJ, Rohde C, Gaskin DJ (2014). The intersection of neighborhood racial segregation, poverty, and urbanicity and its impact on food store availability in the United States. Prev Med.

[5] Bonanno A (2012). Food deserts: demand, supply, and economic theory. Choices..

[6] Bitler M, Haider SJ (2011). An economic view of food deserts in the United States. Journal of Policy Analysis and Management.

[7] Allcott H, Diamond R, Dubé JP, Handbury J, Rahkovsky I, Schnell M (2019). Food deserts and the causes of nutritional inequality. Q J Econ.

[8] Kwate NO (2008). Fried chicken and fresh apples: racial segregation as a fundamental cause of fast food density in black neighborhoods. Health Place.

[9] Thibodeaux J (2016). City racial composition as a predictor of African American food deserts. Urban Stud.

[10] Williams DR, Collins C (2001). Racial residential segregation: a fundamental cause of racial disparities in health. Public Health Rep.

[11] Deener A (2017). The origins of the food desert: urban inequality as infrastructural exclusion. Social Forces.

[12] Thibodeaux J (2016). A historical era of food deserts: changes in the correlates of urban supermarket location, 1970–1990. Social Currents.

[13] Phelan JC, Link BG, Diez-Roux A, Kawachi I, Levin B (2004). “Fundamental causes” of social inequalities in mortality: a test of the theory. J Health Soc Behav.

[14] Feagin JR (2014). Racist America: roots, current realities, and future reparations.

[15] Feagin J (2013). Systemic racism: a theory of oppression.

[16] Massey DS, Denton NA (1988). The dimensions of residential segregation. Social forces.

[17] Kaplan DH (2017). Navigating ethnicity: segregation, placemaking, and difference.

[18] Apparicio P, Martori JC, Pearson AL, Fournier É, Apparicio D (2014). An open-source software for calculating indices of urban residential segregation. Soc Sci Comput Rev.

[19] Florida R, Mellander C (2015). Segregated city: the geography of economic segregation in America’s metros.

[20] Logan JR, Stults BJ. The persistence of segregation in the metropolis: new findings from the 2010 census. Census brief prepared for Project US2010. 2011;24

[21] Gardenhire R, Trapl E, Borawski E (2021). Store classification brief: Cleveland food and tobacco retail project.

[22] US Census Bureau. American Community Survey Design and Methodology (January 2014). Available on-line at: http://www.census.gov/acs/www/Downloads/survey_methodology/acs_design_methodology_report_2014.pdf.

[23] Iceland J (2004). The multigroup entropy index (also known as Theil’s H or the information theory index).

[24] Iceland J, Weinberg DH, Steinmetz E (2002). Racial and ethnic residential segregation in the United States 1980-2000 (Vol. 8, No. 3).

[25] Mitchell A, Griffin LS (2021). The Esri guide to GIS analysis, Volume 2: spatial measurements and statistics (2nd ed.).

[26] James G, Witten D, Hastie T, Tibshirani R (2013). An introduction to statistical learning (Vol. 112, p. 18).

[27] Blangiardo M, Cameletti M (2015). Spatial and spatio-temporal Bayesian models with R-INLA.

[28] Moraga P (2019). Geospatial health data: modeling and visualization with R-INLA and shiny.

[29] Yankey O, Amegbor PM, Essah M (2021). The effect of socioeconomic and environmental factors on obesity: a spatial regression analysis. International Journal of Applied Geospatial Research (IJAGR).

[30] Lunn D, Jackson C, Best N, Thomas A, Spiegelhalter D (2013). The BUGS book. A practical introduction to Bayesian analysis.

[31] Rue H, Martino S, Chopin N (2009). Approximate Bayesian inference for latent Gaussian models by using integrated nested Laplace approximations. Journal of the royal statistical society: Series b (statistical methodology).

[32] Gómez-Rubio V (2020). Bayesian inference with INLA.

[33] Besag J, York J, Mollié A (1991). Bayesian image restoration, with two applications in spatial statistics. Ann Inst Stat Math.

[34] Bakka H, Rue H, Fuglstad GA, Riebler A, Bolin D, Illian J (2018). Spatial modeling with R-INLA: a review. Wiley Interdisciplinary Reviews: Computational Statistics.

[35] Carroll R, Lawson AB, Faes C, Kirby RS, Aregay M, Watjou K (2015). Comparing INLA and OpenBUGS for hierarchical Poisson modeling in disease mapping. Spatial and spatio-temporal epidemiology.

[36] Richardson S, Thomson A, Best N, Elliott P (2004). Interpreting posterior relative risk estimates in disease-mapping studies. Environ Health Perspect.

[37] Lee SY, Song XY (2012). Basic and advanced Bayesian structural equation modeling: with applications in the medical and behavioral sciences.

[38] Spiegelhalter DJ, Best NG, Carlin BP, Van Der Linde A (2002). Bayesian measures of model complexity and fit. Journal of the royal statistical society: Series b (statistical methodology).

[39] Freedman DA, Bell BA, Clark JK, Sharpe PA, Trapl ES, Borawski EA (2019). Socioecological path analytic model of diet quality among residents in two urban food deserts. J Acad Nutr Diet.

[40] Kurtz H. Linking food deserts and racial segregation: challenges and limitations. Geographies of race and food: Fields, bodies, markets. 2013:247–64.

[41] Bellinger WK, Wang J (2011). Poverty, place or race: causes of the retail gap in smaller US cities. The Review of Black Political Economy.

[42] Slocum R, Saldanha A (2013). Geographies of race and food.

[43] Kwate NOA, Yau CY, Loh JM, Williams D (2009). Inequality in obesigenic environments: fast food density in New York City. Health Place.

[44] James P, Arcaya MC, Parker DM, Tucker-Seeley RD, Subramanian SV (2014). Do minority and poor neighborhoods have higher access to fast-food restaurants in the United States?. Health Place.

[45] Scott J, Danos D, Collins R, Simonsen N, Leonardi C, Scribner R, Herd D (2020). Structural racism in the built environment: segregation and the overconcentration of alcohol outlets. Health Place.

[46] Ghosh-Dastidar M, Hunter G, Collins RL, Zenk SN, Cummins S, Beckman R, Nugroho AK, Sloan JC, Dubowitz T (2017). Does opening a supermarket in a food desert change the food environment?. Health Place.

[47] Dubowitz T, Ghosh-Dastidar M, Cohen DA, Beckman R, Steiner ED, Hunter GP, Flórez KR, Huang C, Vaughan CA, Sloan JC, Zenk SN (2015). Diet and perceptions change with supermarket introduction in a food desert, but not because of supermarket use. Health Aff.

[48] Brinkley C, Glennie C, Chrisinger B, Flores J (2019). “If you Build it with them, they will come”: what makes a supermarket intervention successful in a food desert?. J Public Aff.

[49] Howerton G, Trauger A (2017). “Oh honey, don’t you know?” The social construction of food access in a food desert. ACME: An International Journal for Critical Geographies.

[50] Havewala F (2021). The dynamics between the food environment and residential segregation: an analysis of metropolitan areas. Food Policy.

[51] Luan H, Law J, Lysy M (2018). Diving into the consumer nutrition environment: a Bayesian spatial factor analysis of neighborhood restaurant environment. Spatial and spatio-temporal epidemiology.

[52] Lamichhane AP, Warren JL, Peterson M, Rummo P, Gordon-Larsen P (2015). Spatial-temporal modeling of neighborhood sociodemographic characteristics and food stores. Am J Epidemiol.

[53] Liese AD, Barnes TL, Lamichhane AP, Hibbert JD, Colabianchi N, Lawson AB (2013). Characterizing the food retail environment: impact of count, type, and geospatial error in 2 secondary data sources. J Nutr Educ Behav.

