# Supplementary material for: Neighborhood Racial Segregation Predict the Spatial Distribution of Supermarkets and Grocery Stores Better than Socioeconomic Factors in Cleveland, Ohio: a Bayesian Spatial Approach
Source: J Racial Ethn Health Disparities. 2023 Jun 27;11(4):2009–21. doi: 10.1007/s40615-023-01669-4 (PMC11236921; doi:10.1007/s40615-023-01669-4)

**Supplementary Materials**

**Table 6:** Store types and classification by the Prevention Research Center for Healthy Neighborhood (PRCHN)

| **Store Category** | **Criteria** |
| --- | --- |
| Supermarkets (N=12) | - Store has 6 or more point of sale registers - Sells raw meat - In-store bakery - Must sell all of the following:   - Eggs   - Whole wheat/whole grain Bread   - Dried beans   - 2% or whole milk   - 1% or skim milk   - 6 or more varieties of fresh fruit   - 6 or more varieties of fresh vegetables   - 6 or more varieties of processed fruit   - 6 or more varieties of processed vegetables (frozen or canned) |
| Large Grocery (N=47) | • Does not meet all the characteristics of a Supermarket  • Sells raw meat [does not include pre-packaged lunchmeat] OR has an in-store bakery  •Sell 3 or more varieties of EACH of the following:  -Fresh fruit  -Fresh vegetables  -Processed fruit (canned or frozen)  -Processed vegetables (canned or frozen)  • Must sell all of the following:  -Eggs  - Bread (whole wheat/whole grain?)  -Dried beans  -2 or more varieties of milk (whole, 2%, 1% or skim) |
| Small Grocery (N=38) | - Does not meet the characteristics of a Supermarket or a Large Grocery - Must sell at least 1 or more varieties of the following:   - Fresh fruit   - Fresh vegetables   - Processed fruit (canned or frozen)   - Processed vegetables (canned or frozen) - Must sell 2 of the following 5 items:   - Eggs   - Bread (whole wheat/whole grain?)   - Dried beans   - 1 variety of milk   - Raw Meat |
| Cornerstore/Convenience Store(N=520) | - Does not meet the characteristics of a Supermarket, Large or Small Grocery   OR   - Sells food products but sells 0 varieties of at least one of the following:   - Fresh fruit   - Fresh vegetable   - Processed fruit (canned or frozen)   - Processed vegetable (canned or frozen) |
| Fast Food(N=545) | - - Fast-food chain restaurants   - Local fast food retailers   - Takeout |

**Fig 4:** Showing the spatial distribution of the covariates


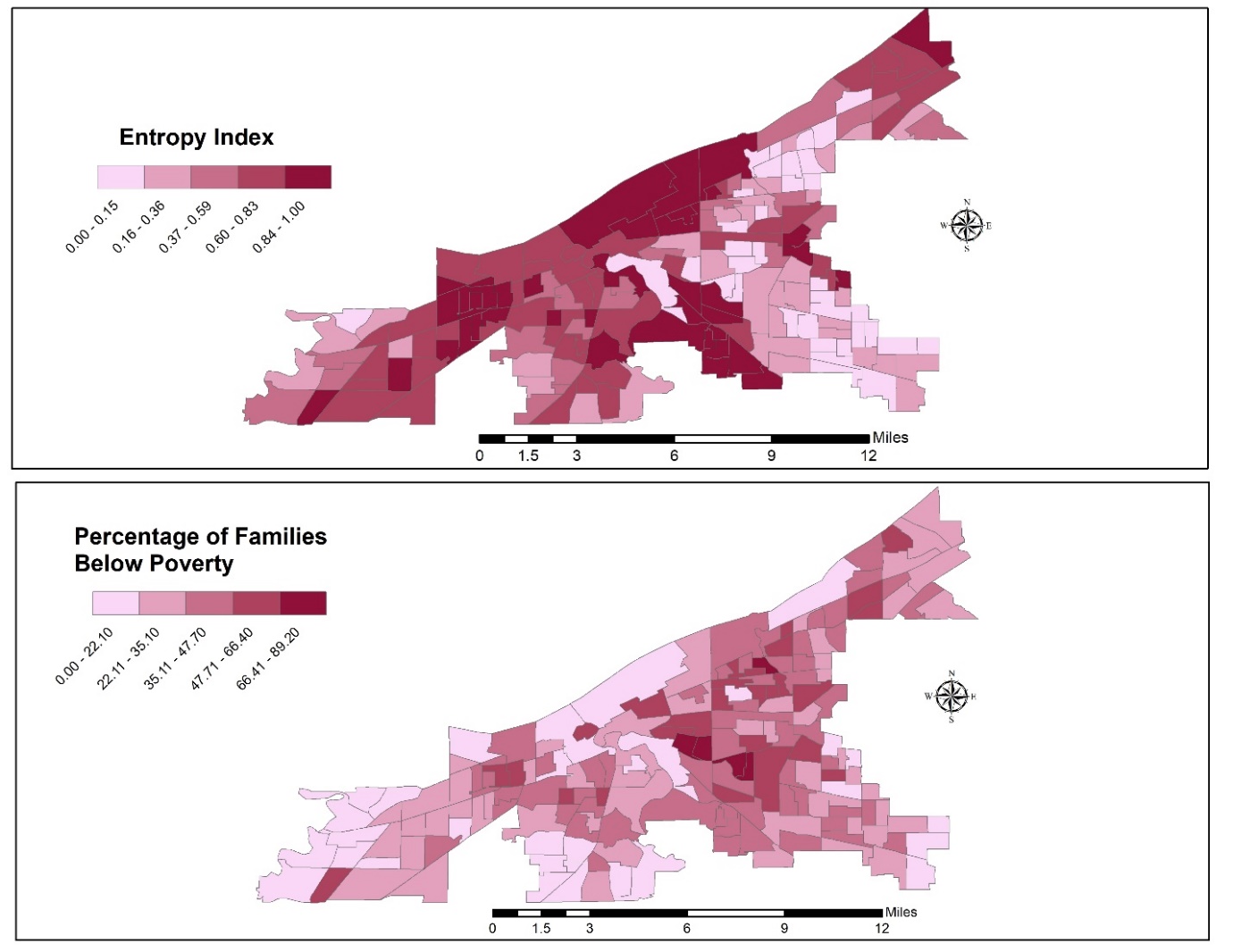


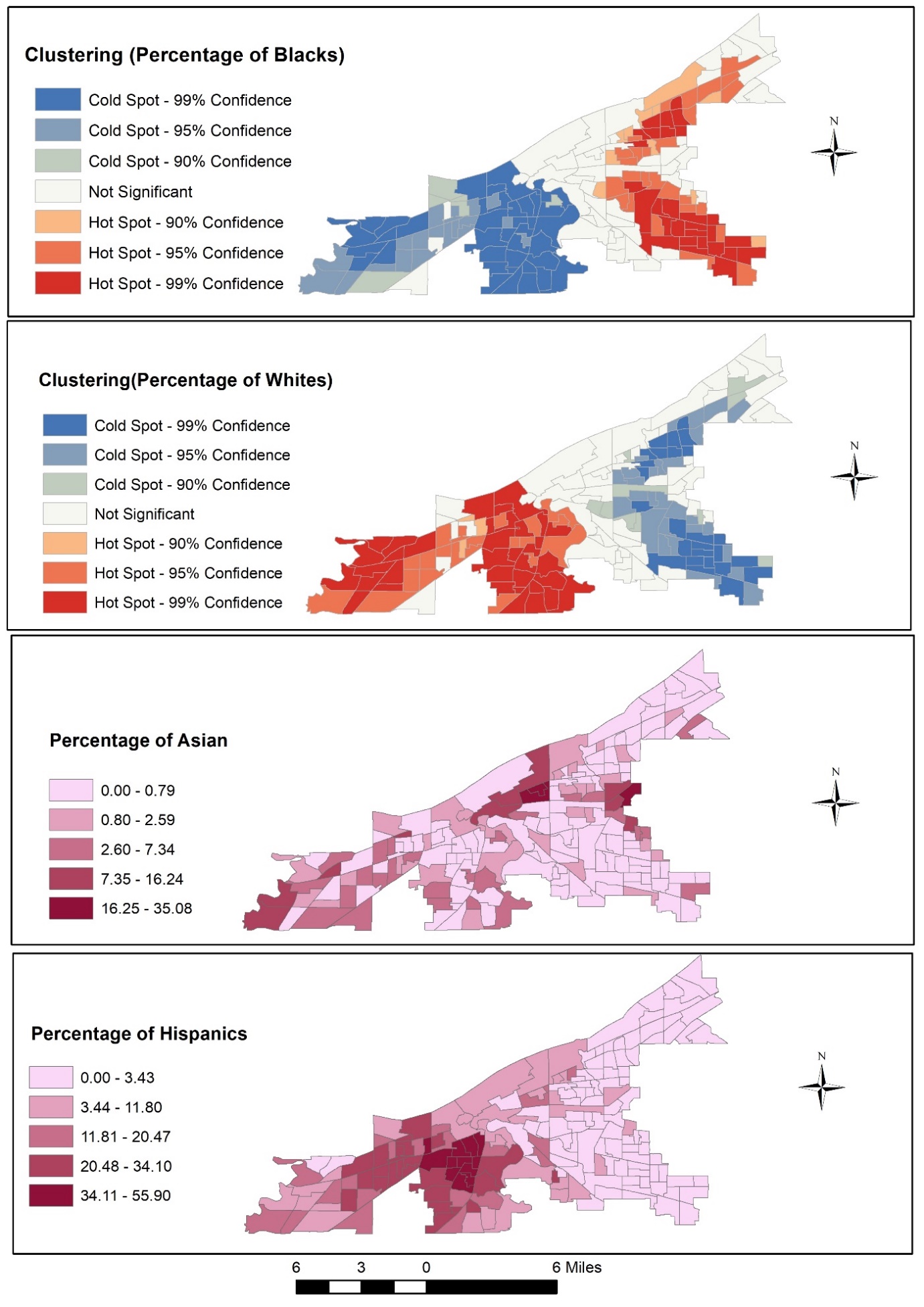


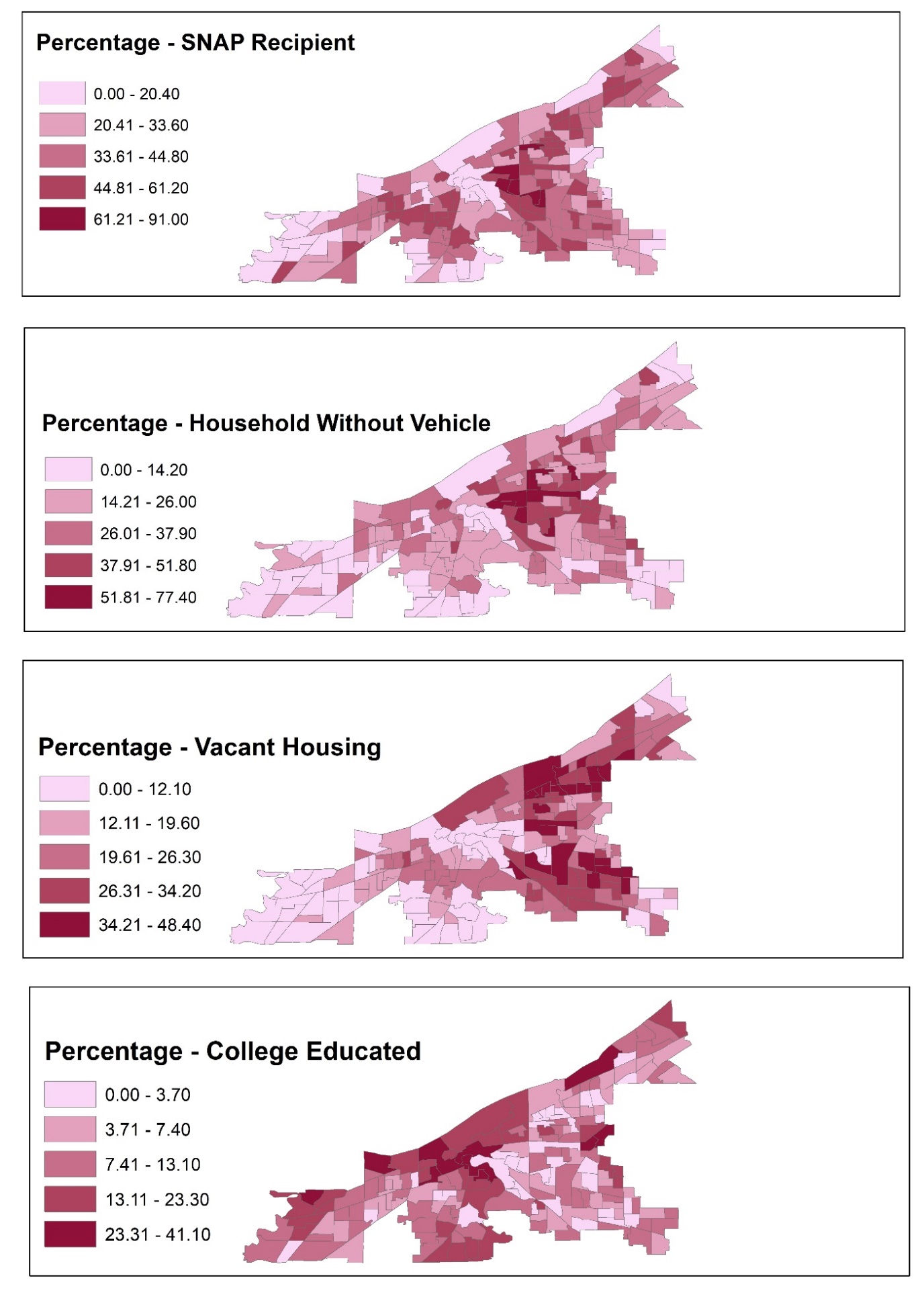


**Fig 5:** Showing the posterior distribution of the covariates in model 1


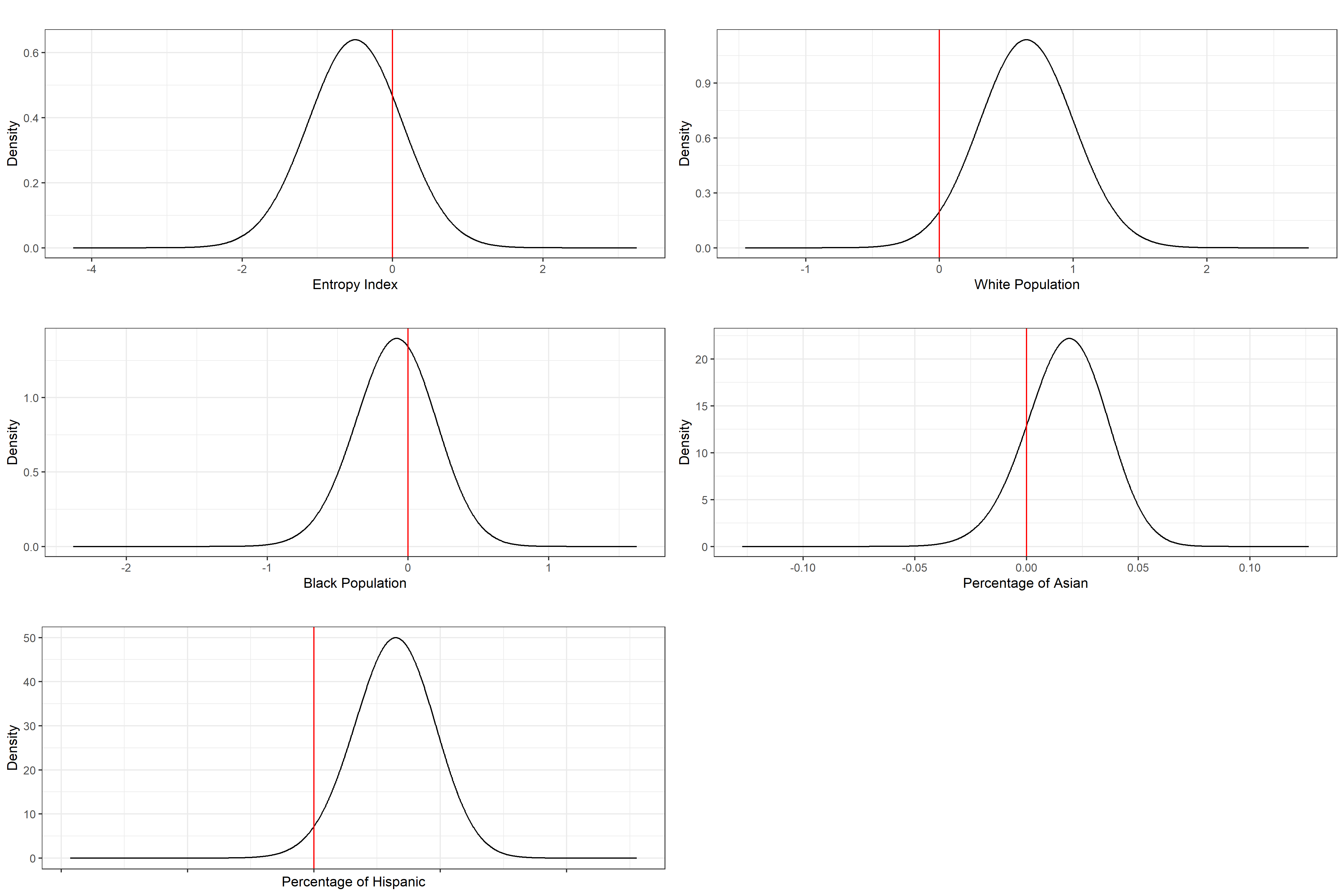


**Fig 6:** Showing the posterior distribution of the covariates in model 2


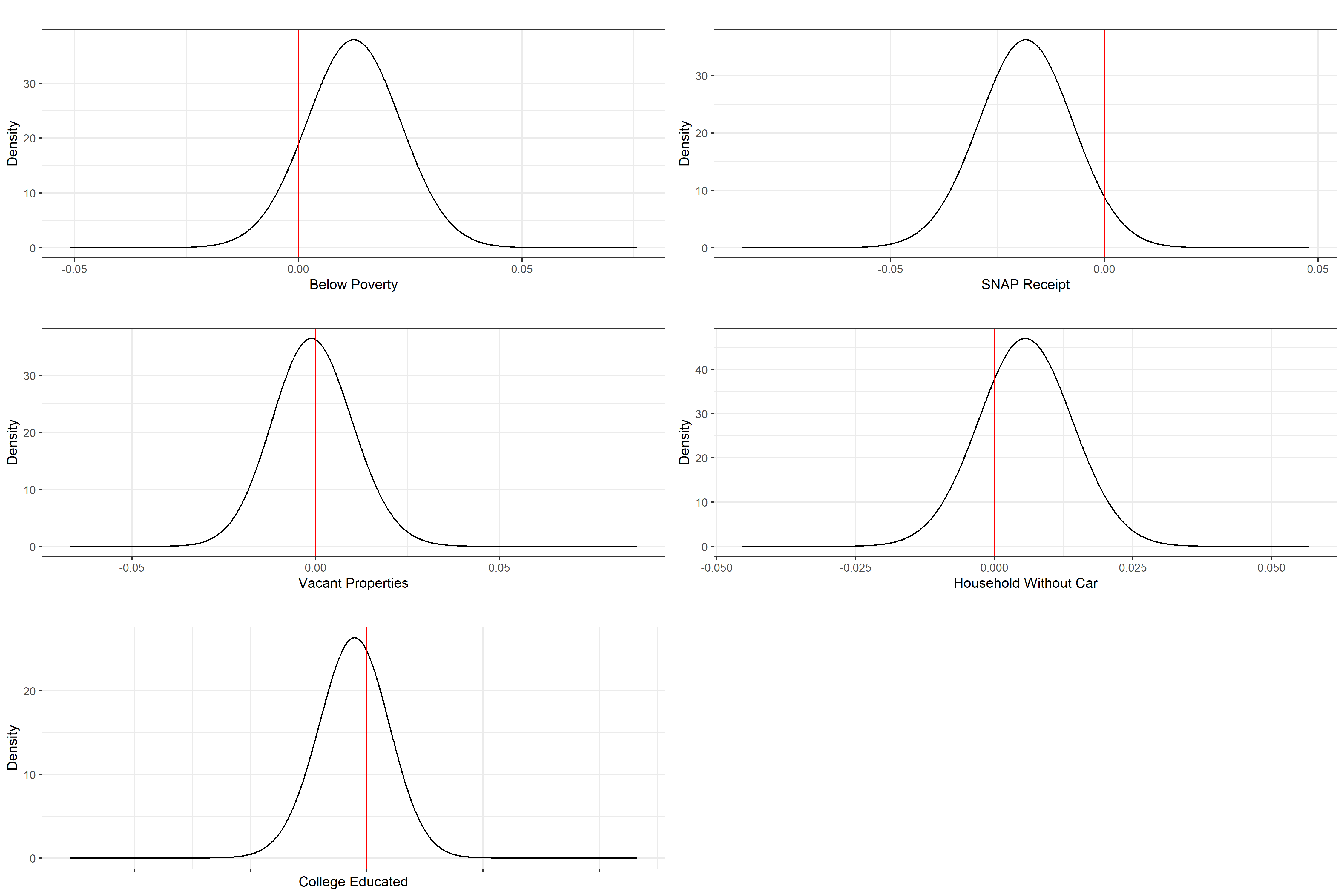


**Fig 7:** Showing the posterior distribution of the covariates in model 3


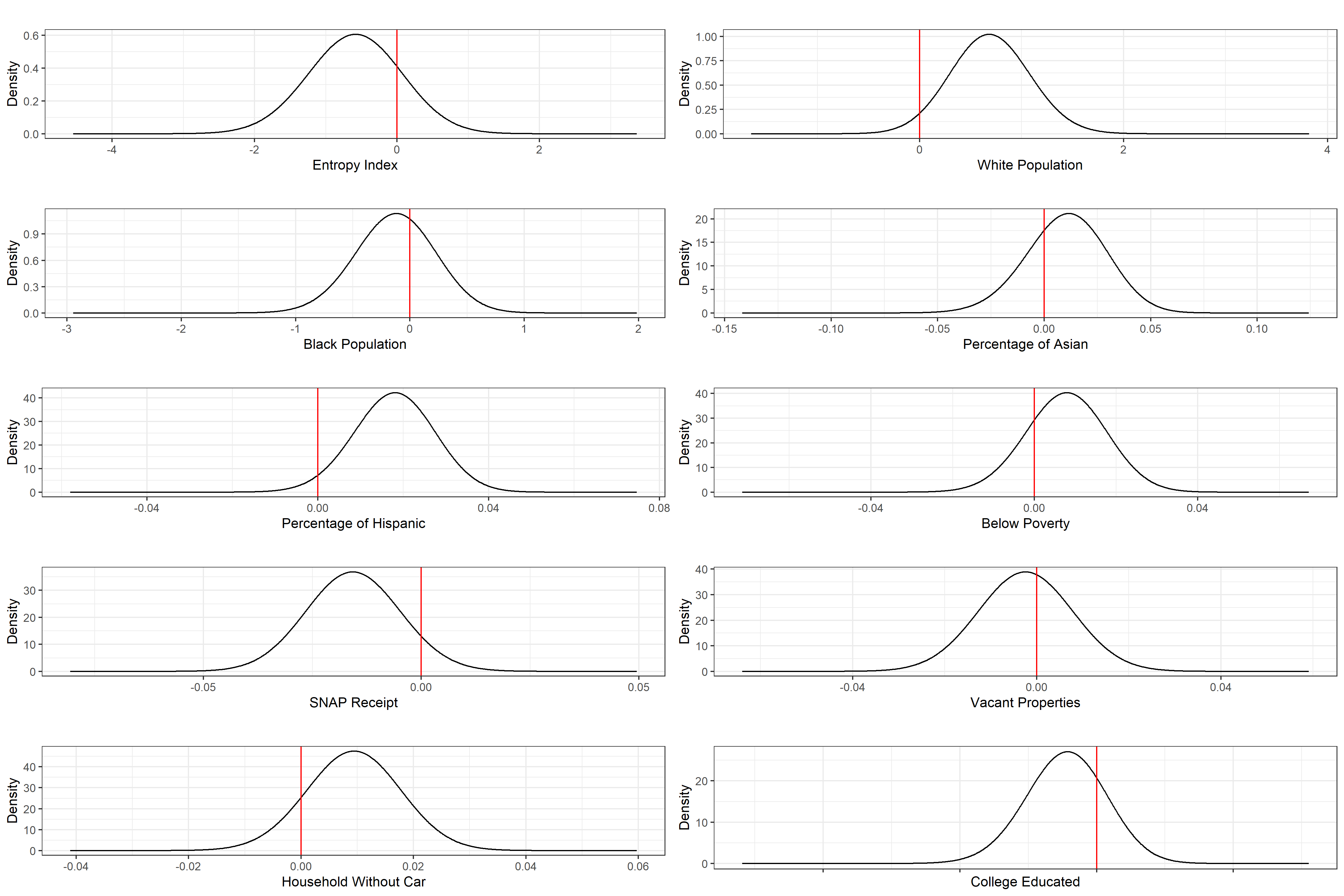

Supplement: Supplementary file 1 — (DOCX 1519 kb) [file 40615_2023_1669_MOESM1_ESM.docx]
